# Survival of cancer survivors with a new pancreatic cancer diagnosis

**DOI:** 10.1002/cam4.4903

**Published:** 2022-06-08

**Authors:** Sandi L. Pruitt, Anna Tavakkoli, Hong Zhu, Daniel F. Heitjan, David E. Gerber, Amit G. Singal, Ethan A. Halm, Muhammad Shaalan Beg, Bhumika Maddineni, Ankit J. Kansagra, Caitlin C. Murphy

**Affiliations:** ^1^ Department of Population & Data Sciences University of Texas Southwestern Medical Center Dallas Texas USA; ^2^ Harold C. Simmons Comprehensive Cancer Center Dallas Texas USA; ^3^ Department of Internal Medicine University of Texas Southwestern Medical Center Dallas Texas USA; ^4^ Department of Statistical Science Southern Methodist University Dallas Texas USA; ^5^ Rutgers Biological Health Sciences (RBHS) Rutgers University New Brunswick New Jersey USA; ^6^ Robert Wood Johnson Medical School Department of Medicine New Brunswick New Jersey USA; ^7^ School of Public Health University of Texas Health Science Center at Houston Houston Texas USA

**Keywords:** clinical trials, multiple malignancies, pancreas cancer, survival

## Abstract

**Background:**

Persons newly diagnosed with pancreas cancer and who have survived a previous cancer are often excluded from clinical trials, despite limited evidence about their prognosis. We examined the association between previous cancer and overall survival.

**Methods:**

This US population‐based cohort study included older adults (aged ≥66 years) diagnosed with pancreas cancer between 2005 and 2015 in the linked Surveillance, Epidemiology, and End Results‐Medicare data. We used Cox proportional hazards models to estimate stage‐specific effects of previous cancer on overall survival, adjusting for sociodemographic, treatment, and tumor characteristics.

**Results:**

Of 32,783 patients, 18.7% were previously diagnosed with another cancer. The most common previous cancers included prostate (29.0%), breast (18.9%), or colorectal (9.7%) cancer. More than half of previous cancers (53.9%) were diagnosed 5 or more years prior to pancreas cancer diagnosis or at an in situ or localized stage (47.8%). The proportions of patients surviving 1, 3, and 5 years after pancreas cancer were nearly identical for those with and without previous cancer. Median survival in months was as follows for those with and without previous cancer respectively: 7 versus 8 (Stage 0/I), 10 versus 10 (Stage II), 7 versus 7 (Stage III), and 3 versus 2 (Stage IV). Cox models indicated that patients with previous cancer had very similar or statistically equivalent survival to those with no previous cancer.

**Conclusions:**

Given nearly equivalent survival compared to those without previous cancer, cancer survivors newly diagnosed with pancreas cancer should be considered for inclusion in pancreas cancer clinical trials.

## Graphical abstract

In this population‐based cohort of 32,783 patients diagnosed with pancreas cancer, nearly one in five were previously diagnosed with another cancer; commonly prostate, breast, or colorectal. Survival was nearly equivalent among those with and without previous cancer.

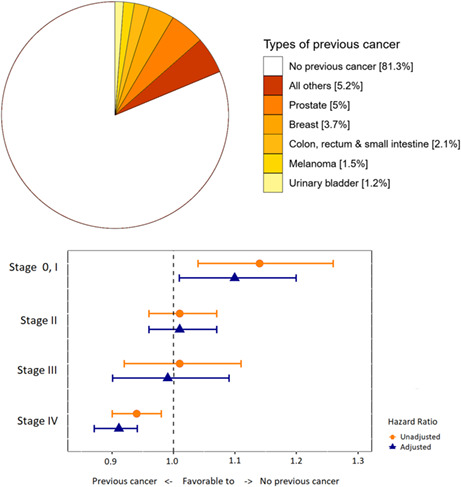

## 1 INTRODUCTION

In 2022, it is estimated that 62,210 people will be diagnosed with and 49,830 people will die from pancreas cancer in the US.^1^ Pancreas cancer is the fourth leading cause of cancer deaths in the US; both incidence and mortality rates have increased since 2000.^2^ Most patients diagnosed with pancreas cancer present with metastatic or unresectable disease, and therefore cytotoxic therapies are the mainstay of treatment.^3^, ^4^ These therapies provide only modest improvements in survival, often measured in weeks to months. As a result, 5‐year relative survival has remained stagnant at about 3% for patients with metastatic disease, in contrast to many other cancers, such as colorectal cancer, for which novel therapies have led to major improvements in survival. However, in recent years, there have been many advancements in adjuvant and neoadjuvant therapies resulting in a marked increase in the number of clinical trials.^3^, ^4^, ^5^, ^6^ Multiple clinical trials are currently enrolling patients to test additional targeted therapies and immunotherapies.^3^, ^4^

For most cancer types, clinical trials exclude cancer survivors newly diagnosed with another cancer,^7^ including the majority of National Cancer Institute (NCI)‐sponsored pancreas cancer trials.^8^, ^9^ Exclusion of this population can hinder accrual efforts for the growing number of clinical trials. A recent systematic review indicates that only 8% of patients with cancer enroll in industry or government‐sponsored clinical trials.^10^ In recent years, multiple organizations have recommended against the reflexive exclusion of cancer survivors. For example, the Food and Drug Administration (FDA) drafted new guidance in 2020 stating that patients with previous or concurrent malignancies should generally be eligible for enrollment in clinical trials.^11^ The American Society of Clinical Oncology–Friends of Cancer Research Clinical Trial Eligibility Working Group similarly recommended against excluding patients solely on the basis of a previous or stable, concurrent malignancy,^12^, ^13^ and the NCI recently issued inclusion criteria template language relating to these recommendations.^14^, ^15^

Excluding patients with pancreas cancer who have survived previous cancer from clinical trials excludes a substantial number of patients, especially older adults, who may benefit from participation in clinical trials and need more effective therapies. A population‐based study from 1975 to 2017 demonstrated that the proportion of patients diagnosed with pancreas cancer and a previous cancer increased from 8.7% in 1975–1984 to 21.9% in 2015–2017.^16^ As the cancer survivor population grows and ages, the number of persons diagnosed with multiple cancers will continue to increase.^17^, ^18^, ^19^ As a consequence, by excluding cancer survivors, clinical trial results are becoming less generalizable to the overall population of people with cancer. Inclusion of patients with previous cancer may also improve the timeliness of trial accrual.

To address this gap, we used US population‐based data of older (age ≥ 65 years) patients newly diagnosed with pancreas cancer to determine whether overall survival differs for patients with previous cancer compared to those without previous cancer. Results will answer critical questions about the prevalence and impact of previous cancer and will inform evidence‐based clinical trial eligibility criteria.

## 2 METHODS

### 2.1 Data

We used linked Medicare claims and National Cancer Institute Surveillance, Epidemiology, and End Results (SEER) program data.^20^ SEER is a nationally representative collection of cancer registries in defined geographic regions and states. Linked SEER‐Medicare data provide additional clinical information on patients enrolled in Medicare. SEER‐Medicare data have been described elsewhere.^20^ For this study, eligible patients included adults diagnosed with pancreas cancer at age ≥ 66 years between 2005 and 2015. We included patients diagnosed with Stage 0–IV pancreas cancer defined using the American Joint Committee on Cancer (*AJCC*) 6th edition; we excluded patients with the unknown stage of pancreas cancer (*n* = 7310). We included patients with common histologic types (see Appendix 1 for codes). To ensure complete data, we included patients with full coverage of Medicare Parts A and B, starting 1 year prior to pancreas cancer diagnosis and continuing at least 1 year after diagnosis or until death. We excluded HMO members, patients with only autopsy or death certificate records, and patients with incomplete dates of diagnosis or death, discrepancies between SEER and Medicare birthdate of 1 year or more, or discrepancies between sequence number and tumor site recode.

### 2.2 Measures

We defined previous cancer using our published approach.^8^, ^21^, ^22^, ^23^ In brief, we determined the order and timing of all primary cancer diagnoses using the SEER variable tumor site recode and associated ICD‐O‐3 values. As described elsewhere,^8^ this approach identifies previous cancers reported to SEER and with a confirmed diagnosis date. We restricted our sample to two groups of patients diagnosed with pancreas cancer: (i) Patients with no previous cancer and (ii) patients with one or more previous cancer of a different type. We excluded the very small number of patients with any previous pancreas cancer (<1%).

We defined overall survival time as the interval in months between pancreas cancer diagnosis (defined as the 15th of the month because SEER provides only month and year of diagnosis) and death from any cause. Patients were followed until death or December 31, 2016.

The following patient characteristics were defined at the time of pancreas cancer diagnosis using SEER data: Age, sex, marital status, race/ethnicity, neighborhood poverty, urban/rural residence, region, and year of diagnosis. Patients with Medicaid were identified using the state buy‐in variable.^24^ We measured the number of 10 frailty‐defining diagnoses (e.g., falls, dementia) and 16 non‐cancer comorbidities (e.g., diabetes, cerebrovascular disease) in the year prior to pancreas cancer diagnosis using Medicare claims (see Appendix 1).^25^, ^26^, ^27^ We measured the following tumor characteristics of the pancreas cancer using SEER data: Tumor location, grade, histology, and stage at diagnosis defined using AJCC, 6th edition. Using both SEER and Medicare claims, we measured receipt of any chemotherapy, radiation, and surgery type. When defining covariates using Medicare claims, we searched inpatient, outpatient, and carrier claims.

### 2.3 Analysis

We reported the prevalence of previous cancer and compared covariates in patients with and without previous cancer using chi‐square tests. We also described the type, timing, and stage of previous cancer.

We conducted survival analyses separately by pancreas cancer stage. First, to illustrate the association of previous cancer and overall survival, we plotted Kaplan–Meier curves and used the log‐rank test to compare overall survival between patients with and without previous cancer. Second, we used Cox proportional hazard models with and without adjustment for all covariates to estimate the hazard rate for overall survival. Hazard ratios (HR) and their 95% confidence intervals (CIs) quantify the effects of previous cancer on overall survival. To assess the proportional hazard assumption, we examined log–log plots and Schoenfeld residuals; when violated, we included interaction terms of previous cancer with (log)time and qualitatively assessed results. Next, to assess whether our findings would be generalizable to a population of patients enrolled in clinical trials, we repeated Cox survival analyses in the subset of patients surviving 30 or more days after their pancreas cancer diagnosis (*n* = 2099, 6.4% of total population). These patients have likely survived long enough to be considered for a clinical trial.

Finally, to better understand absolute differences in survival, we estimated 1‐, 3‐ and 5‐year overall survival for patients with and without previous cancer.

We conducted analyses in SAS 9.4 (SAS Institute Inc.) and created figures in R Studio Version 1.2.5033.^28^

The University of Texas Southwestern Medical Center Institutional Review Board approved this study (STU 042018–032).

## 3 RESULTS

### 3.1 Study population

Appendix 2 shows sample selection. Of 32,783 eligible patients newly diagnosed with pancreas cancer, 18.7% (*n* = 6139) had previous cancer. Table 1 demonstrates differences between those with and without previous cancer. For example, patients with previous cancer were older, more likely to be male and to receive chemotherapy and radiation, more likely to live in lower‐poverty census tracts, and less likely to have Medicaid; further, the proportion of White patients was higher among those with previous cancer. Characteristics of those with and without previous cancer are also shown separately by stage of pancreas cancer in Appendix 3.

**TABLE 1:** Characteristics of patients diagnosed with pancreas cancer, by previous cancer history by analytic cohort

	Overall population				Clinical trial subset of patients surviving >= 30 days			
	No previous cancer(*N* = 26,644)	Previous cancer of other site(*N* = 6139)	*p*‐Value	Total(*N* = 32,783)	No previous cancer(*N* = 1686)	Previous cancer of other site(*N* = 413)	*p*‐Value	Total(*N* = 2099)
Sex								
Male	11,996 (45.0%)	3448 (56.2%)	<0.001	15,444 (47.1%)	765 (45.4%)	226 (54.7%)	<0.001	991 (47.2%)
Female	14,648 (55.0%)	2691 (43.8%)		17,339 (52.9%)	921 (54.6%)	187 (45.3%)		1108 (52.8%)
Age								
66–70	4781 (17.9%)	837 (13.6%)	<0.001	5618 (17.1%)	432 (25.6%)	86.0 (20.8%)	0.0755	518 (24.7%)
70–75	6064 (22.8%)	1299 (21.2%)		7363 (22.5%)	474 (28.1%)	128 (31.0%)		602 (28.7%)
75–80	6002 (22.5%)	1454 (23.7%)		7456 (22.7%)	430 (25.5%)	102 (24.7%)		532 (25.3%)
80–85	5195 (19.5%)	1366 (22.3%)		6561 (20.0%)	243 (14.4%)	76.0 (18.4%)		319 (15.2%)
>85	4602 (17.3%)	1183 (19.3%)		5785 (17.6%)	107 (6.3%)	21.0 (5.1%)		128 (6.1%)
Race/ethnicity								
Non‐Hispanic White	20,671 (77.6%)	5008 (81.6%)	<0.001	25,679 (78.3%)	1353 (80.2%)	336 (81.4%)	0.337	1689 (80.5%)
Hispanic white	1122 (4.2%)	183 (3.0%)		1305 (4.0%)	83.0 (4.9%)	12.0 (2.9%)		95.0 (4.5%)
Black	2560 (9.6%)	540 (8.8%)		3100 (9.5%)	101 (6.0%)	28.0 (6.8%)		129 (6.1%)
Other/Unknown	2291 (8.6%)	408 (6.6%)		2699 (8.2%)	149 (8.8%)	37.0 (9.0%)		186 (8.9%)
Marital status								
Married/Unmarried or domestic partner	13,749 (51.6%)	3433 (55.9%)	<0.001	17,182 (52.4%)	1053 (62.5%)	274 (66.3%)	0.0031	1327 (63.2%)
Separated/Divorced/Widowed	9763 (36.6%)	2018 (32.9%)		11,781 (35.9%)	467 (27.7%)	83.0 (20.1%)		550 (26.2%)
Single	2165 (8.1%)	460 (7.5%)		2625 (8.0%)	114 (6.8%)	34.0 (8.2%)		148 (7.1%)
Unknown	967 (3.6%)	228 (3.7%)		1195 (3.6%)	52.0 (3.1%)	22.0 (5.3%)		74.0 (3.5%)
Medicaid								
Yes	4387 (16.5%)	739 (12.0%)	<0.001	5126 (15.6%)	173 (10.3%)	31.0 (7.5%)	0.109	204 (9.7%)
No	22,257 (83.5%)	5400 (88.0%)		27,657 (84.4%)	1513 (89.7%)	382 (92.5%)		1895 (90.3%)
Poverty								
0% to <10% poverty	13,856 (52.0%)	3397 (55.3%)	<0.001	17,253 (52.6%)	1013 (60.1%)	257 (62.2%)	0.291	1270 (60.5%)
10% to <20% poverty	7529 (28.3%)	1724 (28.1%)		9253 (28.2%)	431 (25.6%)	109 (26.4%)		540 (25.7%)
20% to 100% poverty	5259 (19.7%)	1018 (16.6%)		6277 (19.1%)	242 (14.4%)	47.0 (11.4%)		289 (13.8%)
Urban–rural indicator								
Metro	22,576 (84.7%)	5253 (85.6%)	0.103	27,829 (84.9%)	1464 (86.8%)	357 (86.4%)	0.897	1821 (86.8%)
Non‐metropolitan	4068 (15.3%)	886 (14.4%)		4954 (15.1%)	222 (13.2%)	56.0 (13.6%)		278 (13.2%)
Region								
Northeast	5868 (22.0%)	1281 (20.9%)	<0.001	7149 (21.8%)	430 (25.5%)	111 (26.9%)	0.0102	541 (25.8%)
South	6338 (23.8%)	1142 (18.6%)		7480 (22.8%)	334 (19.8%)	65.0 (15.7%)		399 (19.0%)
Midwest	3265 (12.3%)	1087 (17.7%)		4352 (13.3%)	178 (10.6%)	65.0 (15.7%)		243 (11.6%)
West	11,173 (41.9%)	2629 (42.8%)		13,802 (42.1%)	744 (44.1%)	172 (41.6%)		916 (43.6%)
Grade								
Well differentiated	954 (3.6%)	235 (3.8%)	0.731	1189 (3.6%)	204 (12.1%)	53.0 (12.8%)	0.674	257 (12.2%)
Moderately differentiated	3338 (12.5%)	775 (12.6%)		4113 (12.5%)	550 (32.6%)	142 (34.4%)		692 (33.0%)
Poorly differentiated/Undifferentiated	3612 (13.6%)	846 (13.8%)		4458 (13.6%)	345 (20.5%)	74.0 (17.9%)		419 (20.0%)
Not determined	18,740 (70.3%)	4283 (69.8%)		23,023 (70.2%)	587 (34.8%)	144 (34.9%)		731 (34.8%)
Surgery								
No surgery	22,255 (83.5%)	5077 (82.7%)	0.0876	27,332 (83.4%)	475 (28.2%)	118 (28.6%)	0.986	593 (28.3%)
Whipple	2177 (8.2%)	499 (8.1%)		2676 (8.2%)	589 (34.9%)	143 (34.6%)		732 (34.9%)
Non‐Whipple surgery	2212 (8.3%)	563 (9.2%)		2775 (8.5%)	622 (36.9%)	152 (36.8%)		774 (36.9%)
Chemotherapy								
Yes	11,989 (45.0%)	2991 (48.7%)	<0.001	14,980 (45.7%)	1133 (67.2%)	300 (72.6%)	0.0385	1433 (68.3%)
No	14,655 (55.0%)	3148 (51.3%)		17,803 (54.3%)	553 (32.8%)	113 (27.4%)		666 (31.7%)
Radiation								
Yes	12,337 (46.3%)	3081 (50.2%)	<0.001	15,418 (47.0%)	1180 (70.0%)	305 (73.8%)	0.137	1485 (70.7%)
No	14,307 (53.7%)	3058 (49.8%)		17,365 (53.0%)	506 (30.0%)	108 (26.2%)		614 (29.3%)
Comorbidity count								
0	10,039 (37.7%)	2183 (35.6%)	0.0131	12,222 (37.3%)	770 (45.7%)	189 (45.8%)	0.991	959 (45.7%)
1	8610 (32.3%)	2014 (32.8%)		10,624 (32.4%)	558 (33.1%)	135 (32.7%)		693 (33.0%)
2	4330 (16.3%)	1048 (17.1%)		5378 (16.4%)	236 (14.0%)	60.0 (14.5%)		296 (14.1%)
>= 3	3665 (13.8%)	894 (14.6%)		4559 (13.9%)	122 (7.2%)	29.0 (7.0%)		151 (7.2%)
Frailty count								
0	16,057 (60.3%)	3734 (60.8%)	0.419	19,791 (60.4%)	1110 (65.8%)	298 (72.2%)	0.0359	1408 (67.1%)
1	8404 (31.5%)	1886 (30.7%)		10,290 (31.4%)	510 (30.2%)	105 (25.4%)		615 (29.3%)
>= 2	2183 (8.2%)	519 (8.5%)		2702 (8.2%)	66.0 (3.9%)	10.0 (2.4%)		76.0 (3.6%)
Tumor location								
Pancreas body & tail	6721 (25.2%)	1576 (25.7%)	0.688	8297 (25.3%)	361 (21.4%)	115 (27.8%)	0.00904	476 (22.7%)
Pancreas head	13,367 (50.2%)	3078 (50.1%)		16,445 (50.2%)	1075 (63.8%)	232 (56.2%)		1307 (62.3%)
Pancreas other	6556 (24.6%)	1485 (24.2%)		8041 (24.5%)	250 (14.8%)	66.0 (16.0%)		316 (15.1%)
PC stage								
Stage 0 & 1	2559 (9.6%)	687 (11.2%)	<0.001	3246 (9.9%)	451 (26.7%)	94.0 (22.8%)	0.299	545 (26.0%)
Stage II	6934 (26.0%)	1759 (28.7%)		8693 (26.5%)	942 (55.9%)	247 (59.8%)		1189 (56.6%)
Stage III	2331 (8.7%)	543 (8.8%)		2874 (8.8%)	122 (7.2%)	26.0 (6.3%)		148 (7.1%)
Stage IV	14,820 (55.6%)	3150 (51.3%)		17,970 (54.8%)	171 (10.1%)	46.0 (11.1%)		217 (10.3%)
Vital status								
Alive	1326 (5.0%)	340 (5.5%)	0.0761	1666 (5.1%)	720 (42.7%)	166 (40.2%)	0.384	886 (42.2%)
Dead	25,318 (95.0%)	5799 (94.5%)		31,117 (94.9%)	966 (57.3%)	247 (59.8%)		1213 (57.8%)

Table 2 shows previous cancer type, timing, and stage. For those with more than one previous cancer, *n* = 945 (15.4%) we report characteristics of the most recent previous cancer (relative to the pancreas cancer). The most common previous cancers were prostate (26.5%), breast (19.7%), or colorectal (11.4%). While some previous cancers (14.4%) were diagnosed within 1 year of pancreas cancer, nearly one third (31.7%) were diagnosed within 1–5 years, and the majority (53.9%) were diagnosed more than 5 years before the pancreas cancer. Nearly half of previous cancers (47.8%) were diagnosed at an in situ or localized stage.

**TABLE 2:** Characteristics of previous cancer among patients with pancreas cancer, by analytic cohort

	Overall population					Clinical trial subset of patients surviving >=30 days
	Stage 0 & I *N* = 687 (21.2%)	Stage II *N* = 1759 (20.2%)	Stage III *N* = 543 (18.9%)	Stage IV *N* = 3150 (17.5%)	All stages combined *N* = 6139 (18.7%)	All stages combined *N* = 413
	Number (%)					
Type of previous cancer						
Breast	135 (19.7%)	348 (19.8%)	123 (22.7%)	603 (19.1%)	1209 (19.7%)	78 (18.9%)
Colon; rectum and rectosigmoid junction; Small intestine	79 (11.5%)	180 (10.2%)	74 (13.6%)	365 (11.6%)	698 (11.4%)	40 (9.7%)
Melanoma of the skin; other non‐epithelial skin	45 (6.6%)	148 (8.4%)	33 (6.1%)	279 (8.9%)	505 (8.2%)	29 (7.0%)
Prostate	139 (20.2%)	477 (27.1%)	124 (22.8%)	885 (28.1%)	1625 (26. 5%)	120 (29.0%)
Urinary bladder; ureter; other urinary organs	40 (5.8%)	103 (5.9%)	35 (6.4%)	213 (6.8%)	391 (6.4%)	27 (6.5%)
All others	249 (36.2%)	503 (28.6%)	154 (28.4%)	805 (25.6%)	1711 (27.9%)	119 (28.8%)
Previous cancer timing						
<1 year	134 (19.5%)	254 (14.4%)	73 (13.4%)	420 (13.3%)	881 (14.4)	69 (16.7%)
1–5 years	205 (29.8%)	569 (32.3%)	189 (34.8%)	985 (31.3%)	1948 (31.7)	133 (32.2%)
>5 years	348 (50.7%)	936 (53.2%)	281 (51.7%)	1745 (55.4%)	3310 (53.9)	211 (51.1%)
Stage of previous cancer						
Localized/In‐situ	319 (46.4%)	856 (48.7%)	273 (50.3%)	1485 (47.1%)	2933 (47.8)	184 (44.6%)
Regional	129 (18.8%)	239 (13.6%)	86 (15.8%)	374 (11.9%)	828 (13.5)	59 (14.3%)
Distant	41 (6%)	77 (4.4%)	31 (5.7%)	183 (5.8%)	332 (5.4)	20 (4.8%)
Unknown/missing	198 (28.8%)	587 (33.4%)	153 (28.2%)	1108 (35.2%)	2046 (33.3)	150 (36.3%)

### 3.2 Survival of patients with previous cancer

**FIGURE 1:**
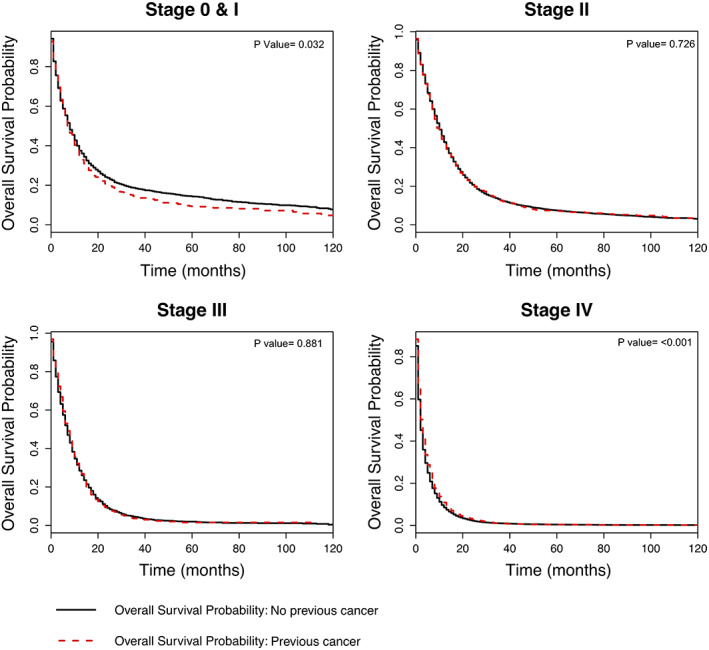
Overall survival curves for pancreas cancer patients with and without previous cancer, by pancreas cancer stage.

Figure 1 illustrates survival curves for patients with and without previous cancer, separately by stage of pancreas cancer. For patients with Stage 0/I pancreas cancer (*n* = 3246), the log‐rank test indicated a statistically significant difference (*p* = 0.032) in overall survival between the two groups, whereby those with previous cancer had worse survival compared to those with no previous cancer. In contrast, for patients with Stage IV pancreas cancer (*n* = 17,970), the survival curve demonstrated that patients with previous cancer had better survival compared to those with no previous cancer; this difference was also statistically significant (*p* < 0.001). There were no statistically significant differences between the two groups for patients with Stage II or III pancreas cancer.

**FIGURE 2:**
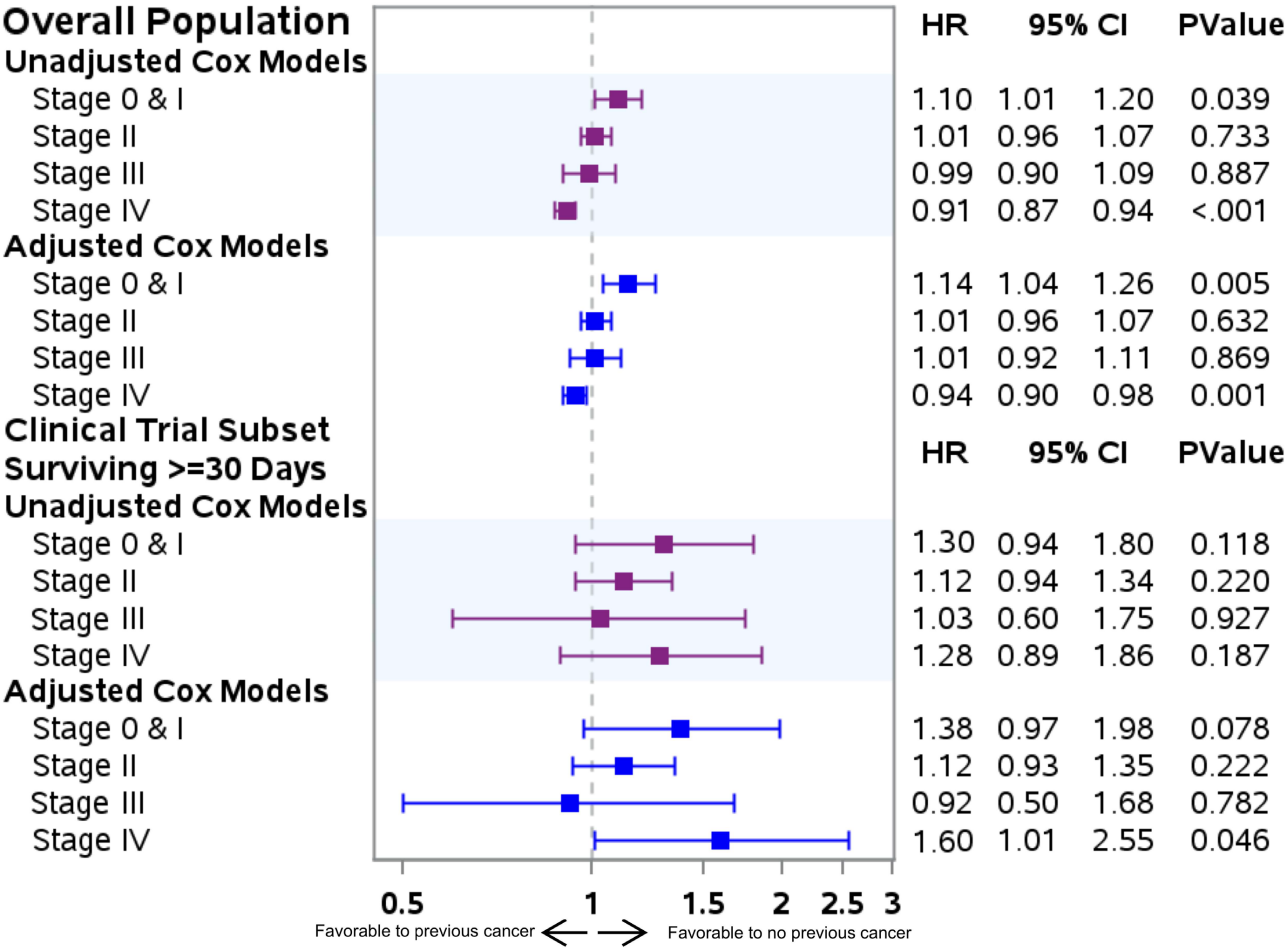
Impact of previous cancer on overall survival among patients with pancreas cancer for the overall population and clinical trial subset surviving ≥30 days after pancreas cancer diagnosis, by pancreas cancer stage.

We tested the proportional hazard assumption in the total study population; it was violated in only two of the eight Cox models (the unadjusted Stage IV model and the adjusted Stage 0/I model). Therefore, to facilitate interpretation and summarize effects, Figure 2 presents the average hazard of previous cancer over time in Cox models without time interactions. Figure 2 also illustrates both the unadjusted and adjusted association of previous cancer and overall survival.

Results from Cox models were consistent with the log‐rank tests. Previous cancer was associated with worse overall survival for patients with Stage 0/I pancreas cancer (adjusted hazard ratio [aHR]: 1.14;95% CI:1.04–1.26) but improved overall survival for patients with Stage IV pancreas cancer (aHR: 0.94;95% CI:0.90–0.98). Patients with Stage II or III pancreas cancer and a previous cancer had equivalent survival to patients with no previous cancer.

### 3.3 1‐, 3‐, and 5‐year survival

To quantify the absolute impact of previous cancer on survival, Table 3 illustrates the proportion of patients alive at 1, 3, and 5 years after diagnosis of pancreas cancer. In general, survival between those with and without previous cancer was equivalent; differences were small, and confidence intervals overlapped in all but three scenarios. First, among patients with Stage IV pancreas cancer, at 1 year after pancreas cancer diagnosis, 11% (95% CI: 10%–12%) of those with previous cancer survived, compared to 8% (95% CI: 8%–9%) of those without previous cancer. Second, among patients with Stage 0/I pancreas cancer, at 3 years after the pancreas cancer diagnosis, 14% (95% CI: 11–17%) of patients with previous cancer were alive compared to 18% (95% CI: 17–20%) of those without. Third, in the same patients with Stage 0/I pancreas cancer, at 5 years after the pancreas cancer diagnosis, 9% (95% CI: 7%–12%) of patients with previous cancer were alive compared to 14% (95% CI: 13%–16%) of those without previous cancer.

**TABLE 3:** Proportions surviving 1‐, 3‐ and 5‐ years after diagnosis of pancreas cancer and median survival time, among the overall population by stage of pancreas cancer

	Stage 0 & 1	Stage II	Stage III	Stage IV
	Months (Interquartile range)			
Median survival				
No previous	8 (3–23)	10 (4–21)	7 (3–14)	2 (1–5)
Previous cancer	7 (3–18)	10 (4–21)	7 (3–17)	3 (1–6)
	Proportion alive (95% confidence interval)			
1‐year survival				
No previous	0.38 (0.36–0.39)	0.43 (0.42–0.44)	0.28 (0.27–0.30)	0.08 (0.08–0.09)
Previous cancer	0.35 (0.31–0.39)	0.42 (0.39–0.44)	0.29 (0.26–0.33)	0.11 (0.10–0.12)
3‐year survival				
No previous	0.18 (0.17–0.20)	0.13 (0.12–0.13)	0.04 (0.04–0.05)	0.01 (0.01–0.01)
Previous cancer	0.14 (0.11–0.17)	0.13 (0.11–0.14)	0.04 (0.02–0.06)	0.009 (0.006–0.01)
5‐year survival				
No previous	0.14 (0.13–0.16)	0.07 (0.07–0.08)	0.02 (0.01–0.03)	0.004 (0.003–0.005)
Previous cancer	0.09 (0.07–0.12)	0.07 (0.06–0.09)	0.01 (0.01–0.03)	0.004 (0.002–0.01)

### 3.4 Subset of patients eligible for clinical trials

Less than 10% of the study population (*n* = 2099) survived 30 or more days after pancreas cancer diagnosis and were included in the subset of patients likely eligible for a clinical trial. Patients included in this subset differed in several ways from the total study population. They were relatively younger, had fewer comorbidities and frailties, and were diagnosed with pancreas cancer at earlier stages and with more favorable clinical characteristics such as tumor grade and location. Of these, 19.7% (*n* = 413) had previous cancer; a percentage similar to that seen in the total population (i.e., 18.7%). There were fewer statistically significant differences in patient characteristics by previous cancer within this subset, possibly due to the smaller sample. One of these differences indicated that, compared to those without previous cancer, those with previous cancer were less likely to have tumors in the head of the pancreas and more likely to have tumors in the body or tail of the pancreas.

There was no statistically significant difference in survival by previous cancer for patients with Stages 0/I, II, or III pancreas cancer (Figure 2). Among patients with Stage IV pancreas cancer, the unadjusted model indicated no difference in survival between those with and without previous cancer; however, the adjusted model indicated that previous cancer was associated with worse overall survival (aHR: 1.60;95% CI: 1.01–2.55).

## 4 DISCUSSION

We observed that nearly one in five (18.7%) patients aged 66 and older who were newly diagnosed with pancreas cancer have survived a previous cancer, and these patients generally have equivalent survival compared to those without previous cancer. There were some exceptions at the extremes of staging and that manifested as small differences in survival. Notably, these small survival differences by previous cancer were largely absent in the subset of patients surviving at least 30 days. In that subset, results indicated that among patients with Stage 0–III pancreas cancer, those with and without previous cancer had equivalent survival. However, among those with Stage IV pancreas cancer, those with previous cancer had worse survival. Based on these findings, we conclude that patients with and without previous cancer generally have equivalent survival. Survival is largely driven by the incident of pancreas cancer and not the previous cancer, likely because pancreas cancer is aggressive and lethal.

One rationale for excluding patients with previous cancer is the potential for previous cancer to negatively impact trial outcomes. There is an implicit assumption that previous cancer leads to worse survival that may negatively impact trial outcomes. However, the limited research about the prognosis of patients with pancreas cancer who have survived previous cancer documents improved survival or no difference. In prior studies, there is some evidence that previous cancer is associated with improved survival among Stage IV patients with pancreatic adenocarcinoma,^29^ a general pancreas cancer population,^30^ and among patients with pancreatic ductal adenocarcinoma^31^; however, another study of patients with pancreatic ductal adenocarcinoma found no difference in overall survival among those with and without previous cancer.^32^ Our study is unique compared to these other studies because we focused on older patients, included patients with all stages of pancreas cancer, and examined stage‐specific patterns. Differences related to eligibility criteria or other methods may account for mixed results in the literature to date.

Several limitations of our study are worth noting. First, some pancreas cancers may represent misclassified metastases from earlier tumors. This is likely rare, however, given strict SEER rules for coding primary cancers^33^ and high rates of microscopic confirmation of SEER‐reported tumors. Another limitation is our lack of data on adults younger than 66 years of age. However, because the prevalence of previous cancer is more common among older versus younger patients with pancreas cancer,^34^ our study reflects the survival of the majority of patients with previous cancer. Third, there are some instances of smaller cell sizes, for example, the clinical trial subset, that may have limited our power to detect differences in survival. Finally, SEER provides only the month and year of diagnosis and therefore our subset analysis may be somewhat imprecise. Among our strengths is our adjustment for important covariates not included in previous studies, such as patient comorbidity and frailty, and our more complete ascertainment of chemotherapy and radiation therapy using data from both SEER and Medicare,^35^ as opposed to previous studies relying on the use of SEER data alone.^29^, ^30^, ^31^, ^32^

We observed some noteworthy differences in our analysis of the total study population versus clinical trial subset surviving 30 days or more. First, in the total population, previous cancer was associated with improved survival for patients with Stage IV cancer; however, these results were reversed in the clinical trial subset. Lead‐time bias may be one explanation. Specifically, if Stage IV pancreas cancer patients with previous cancer experience more frequent or comprehensive medical monitoring and surveillance, their cancers may be diagnosed earlier compared to those without previous cancer. In this scenario, the lead time would result in an artificially prolonged survival (defined as months from date of diagnosis to date of death/censoring) and an apparent survival benefit for patients with previous cancer. However, if lead time is relatively short on average (e.g., a few weeks), this effect would be washed out after limiting to the subset of patients surviving 30 days. Second, in early‐stage patients, previous cancer was associated with worse survival in the total population but was not statistically significantly associated with survival in the clinical trial subset, although the effect estimate was elevated. This might be explained by the smaller size of the subset, resulting in wide confidence intervals and less power to detect modest associations. When assessing results of the subset analysis, it is important to note that the clinical trial subset represents less than 10% of the total population.

### 4.1 Conclusions & implications for clinical trial design

In summary, nearly one in five older patients newly diagnosed with pancreas cancer has survived a previous cancer, and these patients generally have equivalent survival compared to those without previous cancer. Given overall poor survival for patients with pancreas cancer, the clear need for new treatments to improve survival from this aggressive cancer, similar survival of patients regardless of previous cancer history, and the importance of generalizability of clinical trial participants to the larger population, pancreas cancer patients with previous cancer should not be reflexively excluded from clinical trials. Furthermore, a recent study at an academic medical center illustrated that traditionally restrictive eligibility criteria may also perpetuate racial and ethnic disparities in clinical trial enrollment.^37^ The authors illustrated that more inclusive inclusion criteria could equalize trial ineligibility rates between Black and White patients with pancreatic ductal adenocarcinoma, and potentially other cancers.^37^ Importantly, the NCI recently issued guidance and clinical trial template language related to the inclusion of this population in clinical trials.^14^, ^15^ There are recent examples of clinical trial protocols with no explicit exclusion criteria; for example, an NCI network group sponsored trial Alliance A021806 of perioperative versus adjuvant chemotherapy for resectable pancreas cancer.^36^ It will be important to monitor the extent to which additional clinical trials adapt selection criteria accordingly, with the ultimate goal of ensuring the results of clinical trials are generalizable to this growing and under‐studied population.

## AUTHORS' CONTRIBUTIONS

Sandi L. Pruitt designed the study and led the writing; Caitlin C. Murphy provided design and analysis guidance; Anna Tavakkoli provided code and subject matter expertise. Hong Zhu, Daniel F. Heitjan, and Bhumika Maddineni designed, critically evaluated, and/or performed data analysis. David E. Gerber, Anna Tavakkoli, Muhammad Shaalan Beg, Ethan A. Halm, Ankit J. Kansagra and Amit G. Singal provided clinical insights. All authors interpreted the results and provided critical feedback and edits to the manuscript and approved the final manuscript.

## FUNDING INFORMATION

National Cancer Institute R01CA229834 (SLP) and K24CA201543 (DEG).

## CONFLICT OF INTEREST

Daniel F. Heitjan reports consulting (Bluejay Diagnostics, Braintree labs, Creatics LLC, Abbott Labs, Macrogenics, Inc.,) and legal consulting (Noven, Pfizer, Women's Talc Project); Caitlin C. Murphy reports consulting (Freenome); Amit G. Singal reports consulting/advisory boards (Bayer, Eisai, Genentech, BMS, Exelixis, Exact Sciences, GRAIL); David E. Gerber reports research funding (Astra‐Zeneca, BerGenBio, Karyopharm), stock ownership (Gilead) and consulting (Samsung Bioepis, Jansen, Catalyst Pharmaceuticals). Ankit J. Kansagra reports advisory board (Alynylam, BMS/Celgene, Cota Health, GSK, Janssen, Oncopeptide).

## Supporting information


Appendix S1‐S3

## Data Availability

This study used SEER‐MEDICARE data. The Centers for Medicare and Medicaid Services do not allow the redistribution of their data by researchers. SEER‐MEDICARE data are distinct from the publicly available SEER database, and can be obtained by researchers, by following the process described on https://healthcaredelivery.cancer.gov/seermedicare/obtain/requests.html (access requirements include Institutional Review Board approval and the completion of a Data Use Agreement). Code available upon request.
