# Supplementary material for: Survival of cancer survivors with a new pancreatic cancer diagnosis
Source: Cancer Med. 2022 Jun 8;12(1):200–12. doi: 10.1002/cam4.4903 (PMC9844592; doi:10.1002/cam4.4903)
Supplement: Supplementary file 1 — Appendix S1‐S3 [file CAM4-12-200-s001.docx]

*Appendix 1 Comorbid conditions, frailty-defining diagnoses and histology types included in the study sample*

| Comorbid conditions |
| --- |
| Acute Myocardial Infarction |
| History of Myocardial Infarction |
| Congestive Heart Failure |
| Peripheral Vascular Disease |
| Cerebrovascular Disease |
| Chronic Obstructive Pulmonary Disease |
| Dementia |
| Hemiplegia or Paraplegia |
| Diabetes |
| Diabetes with Complications |
| Moderate-Severe Renal Disease |
| Mild Liver Disease |
| Moderate-Severe Liver Disease |
| Peptic Ulcer Disease |
| Rheumatologic Disease |
| Acquired Immunodeficiency Virus (AIDS) |
| Frailty-defining diagnoses |
| Malnutrition: Nutritional marasmus; Other severe protein-calorie malnutrition |
| Dementia: Senile dementia with delusional or depressive features; Senile dementia with delirium |
| Severe vision impairment: Profound impairment, both eyes; Moderate or severe impairment, better eye/lesser eye: profound |
| Decubitus ulcer |
| Incontinence of urine: Incontinence without sensory awareness; Continuous leakage |
| Loss of weight: Abnormal loss of weight and underweight; Feeding difficulties and mismanagement |
| Fecal incontinence: Incontinence of feces |
| Social support needs: Lack of housing; Inadequate housing; Inadequate material resources |
| Difficulty in walking: Difficulty in walking; Abnormality of gait |
| Fall: Fall on stairs or steps, Fall from wheelchair |
| Included histology codes |
| 8000- Neoplasm, Malignant |
| 8010- Carcinoma, In situ NOS |
| 8020- Carcinoma, undifferentiated type, NOS |
| 8021- Carcinoma, anaplastic type, NOS |
| 8211- Tubular adenocarcinoma |
| 8140- Adenocarcinoma in situ |
| 8230- Duct carcinoma in situ, solid type |
| 8453- Intraductal papillary-mucinous carcinoma, non-inv. |
| 8470 and 8472-Mucinous cystadenocarcinoma, NOS |
| 8480-Mucinous adenocarcinoma |
| 8481-Mucin-producing adenocarcinoma |
| 8022-Pleomorphic carcinoma |
| 8503-Intraductal papillary adenocarcinoma with invasion |
| 8141-Scirrhous adenocarcinoma |
| 8570- Adenocarcinoma with squamous metaplasia |
| 8521-Infiltrating ductular carcinoma |
| 8550-Acinar cell carcinoma |
| 8500-Intraductal carcinoma, noninfiltrating; Infiltrating duct carcinoma, NOS |
| 8560-Adenosquamous carcinoma |

*Appendix 2 Diagram of patient selection from Surveillance, Epidemiology, and End Results (SEER)-Medicare data of patients diagnosed with pancreas cancer, 2005-2015*

N=3048 excluded due to unknown sequence number and reporting on death certificate or autopsy only

N=14618 excluded due to age <66 years at diagnosis

N=25596 excluded without at least 12 months continuous Parts A&B, non-HMO enrollment prior to diagnosis and at least 12 months/ maximum continuous Parts A&B, non-HMO enrollment after diagnosis

N=3798 excluded due to with a sequence number suggesting prior cancer that was not SEER-confirmed by SEER site recode variable

N=842 excluded with discrepancies in SEER and Medicare birth dates of a year or more and due to unknown death month or year in SEER

N=89478 pancreas cancer cases

N=**33354** included

N=5470 One prior of other site

N=27884 No prior

Overall: N=32,783 (N=6,139, 18.7% with previous cancer);

Subset surviving ≥30 days: N = 2,099 (N= 413, 19.7% with previous cancer)

N=1987 excluded due to restricted histology codes and having or >=1 prior of same site*

N=48 excluded due to unknown poverty and/or due to unknown urban-rural indicator

N=7310 excluded due to unknown stage

*Appendix 3. Characteristics of patients diagnosed with pancreas cancer by previous cancer history and stage of pancreas cancer, among the overall study population*

|  | **Stage 0 & 1** | | **Stage II** | | **Stage III** | | **Stage IV** | | |
| --- | --- | --- | --- | --- | --- | --- | --- | --- | --- |
|  | **No previous cancer** | **Previous cancer of other site** | **No previous cancer** | **Previous cancer of other site** | **No previous cancer** | **Previous cancer of other site** | **No previous cancer** | **Previous cancer of other site** |  |
|  | **(N=2559)** | **(N=687)** | **(N=6934)** | **(N=1759)** | **(N=2331)** | **(N=543)** | **(N=14820)** | **(N=3150)** |  |
| **Sex** |  |  |  |  |  |  |  |  |  |
| Male | 1068 (41.7%) | 352 (51.2%) | 3093 (44.6%) | 961 (54.6%) | 1029 (44.1%) | 289 (53.2%) | 6806 (45.9%) | 1846 (58.6%) |  |
| Female | 1491 (58.3%) | 335 (48.8%) | 3841 (55.4%) | 798 (45.4%) | 1302 (55.9%) | 254 (46.8%) | 8014 (54.1%) | 1304 (41.4%) |  |
| **Age** |  |  |  |  |  |  |  |  |  |
| 66-70 | 306 (12.0%) | 66 (9.6%) | 1341 (19.3%) | 248 (14.1%) | 481 (20.6%) | 88 (16.2%) | 2653 (17.9%) | 435 (13.8%) |  |
| 70-75 | 430 (16.8%) | 114 (16.6%) | 1654 (23.9%) | 405 (23.0%) | 566 (24.3%) | 118 (21.7%) | 3414 (23.0%) | 662 (21.0%) |  |
| 75-80 | 531 (20.8%) | 149 (21.7%) | 1639 (23.6%) | 408 (23.2%) | 562 (24.1%) | 127 (23.4%) | 3270 (22.1%) | 770 (24.4%) |  |
| 80-85 | 570 (22.3%) | 161 (23.4%) | 1355 (19.5%) | 405 (23.0%) | 407 (17.5%) | 121 (22.3%) | 2863 (19.3%) | 679 (21.6%) |  |
| >85 | 722 (28.2%) | 197 (28.7%) | 945 (13.6%) | 293 (16.7%) | 315 (13.5%) | 89 (16.4%) | 2620 (17.7%) | 604 (19.2%) |  |
| **Race/ethnicity** |  |  |  |  |  |  |  |  |  |
| Non-Hispanic White | 1993 (77.9%) | 552 (80.3%) | 5538 (79.9%) | 1465 (83.3%) | 1774 (76.1%) | 434 (79.9%) | 11366 (76.7%) | 2557 (81.2%) |  |
| Hispanic white | 101 (3.9%) | 22 (3.2%) | 279 (4.0%) | 46 (2.6%) | 94 (4.0%) | 21 (3.9%) | 648 (4.4%) | 94 (3.0%) |  |
| Black | 236 (9.2%) | 60 (8.7%) | 542 (7.8%) | 139 (7.9%) | 228 (9.8%) | 52 (9.6%) | 1554 (10.5%) | 289 (9.2%) |  |
| Other/Unknown | 229 (8.9%) | 53 (7.7%) | 575 (8.3%) | 109 (6.2%) | 235 (10.1%) | 36 (6.6%) | 1252 (8.4%) | 210 (6.7%) |  |
| **Marital status** |  |  |  |  |  |  |  |  |  |
| Married/Unmarried or domestic partner | 1196 (46.7%) | 347 (50.5%) | 3825 (55.2%) | 1006 (57.2%) | 1251 (53.7%) | 313 (57.6%) | 7477 (50.5%) | 1767 (56.1%) |  |
| Separated/Divorced/Widowed | 1063 (41.5%) | 252 (36.7%) | 2330 (33.6%) | 559 (31.8%) | 819 (35.1%) | 176 (32.4%) | 5551 (37.5%) | 1031 (32.7%) |  |
| Single | 205 (8.0%) | 65 (9.5%) | 505 (7.3%) | 130 (7.4%) | 196 (8.4%) | 33 (6.1%) | 1259 (8.5%) | 232 (7.4%) |  |
| Unknown | 95 (3.7%) | 23 (3.3%) | 274 (4.0%) | 64 (3.6%) | 65 (2.8%) | 21 (3.9%) | 533 (3.6%) | 120 (3.8%) |  |
| **Medicaid** |  |  |  |  |  |  |  |  |  |
| Yes | 475 (18.6%) | 135 (19.7%) | 978 (14.1%) | 184 (10.5%) | 338 (14.5%) | 62 (11.4%) | 2596 (17.5%) | 358 (11.4%) |  |
| No | 2084 (81.4%) | 552 (80.3%) | 5956 (85.9%) | 1575 (89.5%) | 1993 (85.5%) | 481 (88.6%) | 12224 (82.5%) | 2792 (88.6%) |  |
| **Poverty** |  |  |  |  |  |  |  |  |  |
| 0%-<10% poverty | 1224 (47.8%) | 338 (49.2%) | 3766 (54.3%) | 995 (56.6%) | 1244 (53.4%) | 327 (60.2%) | 7622 (51.4%) | 1737 (55.1%) |  |
| 10% to <20% poverty | 777 (30.4%) | 212 (30.9%) | 1875 (27.0%) | 487 (27.7%) | 658 (28.2%) | 139 (25.6%) | 4219 (28.5%) | 886 (28.1%) |  |
| 20% to 100% poverty | 558 (21.8%) | 137 (19.9%) | 1293 (18.6%) | 277 (15.7%) | 429 (18.4%) | 77 (14.2%) | 2979 (20.1%) | 527 (16.7%) |  |
| **Urban-rural indicator** |  |  |  |  |  |  |  |  |  |
| Metro | 2131 (83.3%) | 573 (83.4%) | 5893 (85.0%) | 1513 (86.0%) | 1978 (84.9%) | 471 (86.7%) | 12574 (84.8%) | 2696 (85.6%) |  |
| Non-metropolitan | 428 (16.7%) | 114 (16.6%) | 1041 (15.0%) | 246 (14.0%) | 353 (15.1%) | 72 (13.3%) | 2246 (15.2%) | 454 (14.4%) |  |
| **Region** |  |  |  |  |  |  |  |  |  |
| Northeast | 469 (18.3%) | 120 (17.5%) | 1523 (22.0%) | 406 (23.1%) | 553 (23.7%) | 128 (23.6%) | 3323 (22.4%) | 627 (19.9%) |  |
| South | 716 (28.0%) | 139 (20.2%) | 1634 (23.6%) | 338 (19.2%) | 528 (22.7%) | 106 (19.5%) | 3460 (23.3%) | 559 (17.7%) |  |
| Midwest | 286 (11.2%) | 117 (17.0%) | 810 (11.7%) | 307 (17.5%) | 259 (11.1%) | 84 (15.5%) | 1910 (12.9%) | 579 (18.4%) |  |
| West | 1088 (42.5%) | 311 (45.3%) | 2967 (42.8%) | 708 (40.3%) | 991 (42.5%) | 225 (41.4%) | 6127 (41.3%) | 1385 (44.0%) |  |
| **Grade** |  |  |  |  |  |  |  |  |  |
| Well differentiated | 154 (6.0%) | 38 (5.5%) | 452 (6.5%) | 119 (6.8%) | 120 (5.1%) | 22 (4.1%) | 228 (1.5%) | 56 (1.8%) |  |
| Moderately differentiated | 332 (13.0%) | 85 (12.4%) | 1788 (25.8%) | 447 (25.4%) | 315 (13.5%) | 54 (9.9%) | 903 (6.1%) | 189 (6.0%) |  |
| Poorly differentiated/Undifferentiated | 249 (9.7%) | 73 (10.6%) | 1587 (22.9%) | 376 (21.4%) | 293 (12.6%) | 61 (11.2%) | 1483 (10.0%) | 336 (10.7%) |  |
| Not determined | 1824 (71.3%) | 491 (71.5%) | 3107 (44.8%) | 817 (46.4%) | 1603 (68.8%) | 406 (74.8%) | 12206 (82.4%) | 2569 (81.6%) |  |
| **Surgery** |  |  |  |  |  |  |  |  |  |
| No surgery | 1924 (75.2%) | 535 (77.9%) | 3624 (52.3%) | 952 (54.1%) | 2140 (91.8%) | 501 (92.3%) | 14567 (98.3%) | 3089 (98.1%) |  |
| Whipple | 247 (9.7%) | 50 (7.3%) | 1737 (25.1%) | 407 (23.1%) | 98 (4.2%) | 24 (4.4%) | 95 (0.6%) | 18 (0.6%) |  |
| Non-Whipple surgery | 388 (15.2%) | 102 (14.8%) | 1573 (22.7%) | 400 (22.7%) | 93 (4.0%) | 18 (3.3%) | 158 (1.1%) | 43 (1.4%) |  |
| **Chemotherapy** |  |  |  |  |  |  |  |  |  |
| Yes | 808 (31.6%) | 247 (36.0%) | 3979 (57.4%) | 999 (56.8%) | 1356 (58.2%) | 330 (60.8%) | 5846 (39.4%) | 1415 (44.9%) |  |
| No | 1751 (68.4%) | 440 (64.0%) | 2955 (42.6%) | 760 (43.2%) | 975 (41.8%) | 213 (39.2%) | 8974 (60.6%) | 1735 (55.1%) |  |
| **Radiation** |  |  |  |  |  |  |  |  |  |
| Yes | 1068 (41.7%) | 343 (49.9%) | 3994 (57.6%) | 1045 (59.4%) | 1380 (59.2%) | 342 (63.0%) | 5895 (39.8%) | 1351 (42.9%) |  |
| No | 1491 (58.3%) | 344 (50.1%) | 2940 (42.4%) | 714 (40.6%) | 951 (40.8%) | 201 (37.0%) | 8925 (60.2%) | 1799 (57.1%) |  |
| **Comorbidity count** |  |  |  |  |  |  |  |  |  |
| 0 | 837 (32.7%) | 189 (27.5%) | 2676 (38.6%) | 652 (37.1%) | 931 (39.9%) | 206 (37.9%) | 5595 (37.8%) | 1136 (36.1%) |  |
| 1 | 811 (31.7%) | 232 (33.8%) | 2277 (32.8%) | 594 (33.8%) | 815 (35.0%) | 184 (33.9%) | 4707 (31.8%) | 1004 (31.9%) |  |
| 2 | 434 (17.0%) | 122 (17.8%) | 1102 (15.9%) | 294 (16.7%) | 349 (15.0%) | 85 (15.7%) | 2445 (16.5%) | 547 (17.4%) |  |
| >= 3 | 477 (18.6%) | 144 (21.0%) | 879 (12.7%) | 219 (12.5%) | 236 (10.1%) | 68 (12.5%) | 2073 (14.0%) | 463 (14.7%) |  |
| **Frailty count** |  |  |  |  |  |  |  |  |  |
| 0 | 1467 (57.3%) | 416 (60.6%) | 4275 (61.7%) | 1072 (60.9%) | 1292 (55.4%) | 297 (54.7%) | 9023 (60.9%) | 1949 (61.9%) |  |
| 1 | 818 (32.0%) | 203 (29.5%) | 2128 (30.7%) | 540 (30.7%) | 868 (37.2%) | 195 (35.9%) | 4590 (31.0%) | 948 (30.1%) |  |
| >=2 | 274 (10.7%) | 68 (9.9%) | 531 (7.7%) | 147 (8.4%) | 171 (7.3%) | 51 (9.4%) | 1207 (8.1%) | 253 (8.0%) |  |
| **Vital status** |  |  |  |  |  |  |  |  |  |
| Alive | 366 (14.3%) | 82 (11.9%) | 689 (9.9%) | 187 (10.6%) | 99 (4.2%) | 26 (4.8%) | 172 (1.2%) | 45 (1.4%) |  |
| Dead | 2193 (85.7%) | 605 (88.1%) | 6245 (90.1%) | 1572 (89.4%) | 2232 (95.8%) | 517 (95.2%) | 14648 (98.8%) | 3105 (98.6%) |  |
